# In this issue: Volume 114, Issue 2, February 2023

**DOI:** 10.1111/cas.15406

**Published:** 2023-02-05

## TAS2940, A novel brain‐penetrable PAN‐ERBB inhibitor, for tumors with HER2 and EGFR aberrations


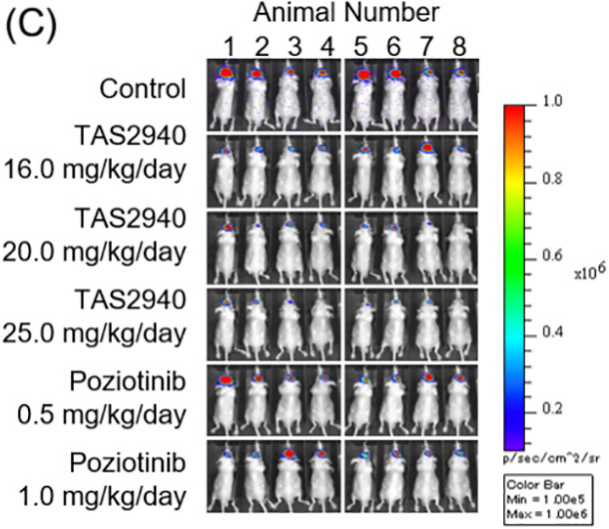


The epidermal growth factor receptor (EGFR) is a member of the avian erythroblastosis oncogene B (ERBB) family, and is crucial for mammalian cell growth and multiplication. Mutations in the human epidermal growth factor receptor type 2 (*HER2*) are commonly associated with fast‐spreading cancers, including aggressive brain metastatic tumors.

Non‐small‐cell lung cancers (NSCLCs) containing unique *HER2* exon 20 insertion mutations make them resistant to conventional cancer treatments. Notably, a substantial number of patients with NSCLC and breast cancer develop metastases wherein the cancer spreads to their brain. Unfortunately, no pan‐ERBB inhibitors that can penetrate the blood brain barrier are currently available. As a result, it is critical to develop pan‐ERBB inhibitors that can penetrate the brain and are active against aberrant HER2/EGFR.

In this issue, Oguchi et al. evaluated the efficacy of a new, irreversible pan‐ERBB inhibitor—TAS2940 fumarate (hereafter TAS2940)—and its ability to penetrate the blood–brain barrier. They found that TAS2940 was highly potent against cancer cells harboring mutations in the *HER2/EGFR* gene. It could selectively inhibit the growth of these cells by preventing their phosphorylation. Enzymatic assays indicated that TAS2940 was highly selective in inhibiting EGFRs as compared to poziotinib—a widely used ERBB inhibitor. This selectivity would render TAS2940 less toxic to cells, making it more desirable for use in clinical trials.

In addition, they found that TAS2940 not only reduced tumor growth in cancer cell lines but also in mice models with no immunity, which were injected with human cancer cells. The inhibitory effects of TAS2940 were similar to those of poziotinib.

Surprisingly, treatment with TAS2940 reduced tumor growth more effectively than that with poziotinib in a mice intracranial model. The most remarkable finding was that TAS2940 could penetrate the blood–brain barrier. Of major HER2/EGFR inhibitors examined, it exhibited the strongest penetrative ability, with the exception of osimertinib.

To summarize, TAS2940 is a highly promising solution for aggressive cancers with *ERBB* mutations. It is now a proven brain‐penetrable pan‐ERBB inhibitor with potent antitumor effects against a wide range of cancers. These findings, together with the ongoing clinical trials, provide strong rationale for evaluating TAS2940 in patients with *HER2*/*EGFR* aberrations and brain metastases. https://onlinelibrary.wiley.com/doi/full/10.1111/cas.15617

## Engulfment and cell motility protein 1 fosters reprogramming of tumor‐associated macrophages in colorectal cancer


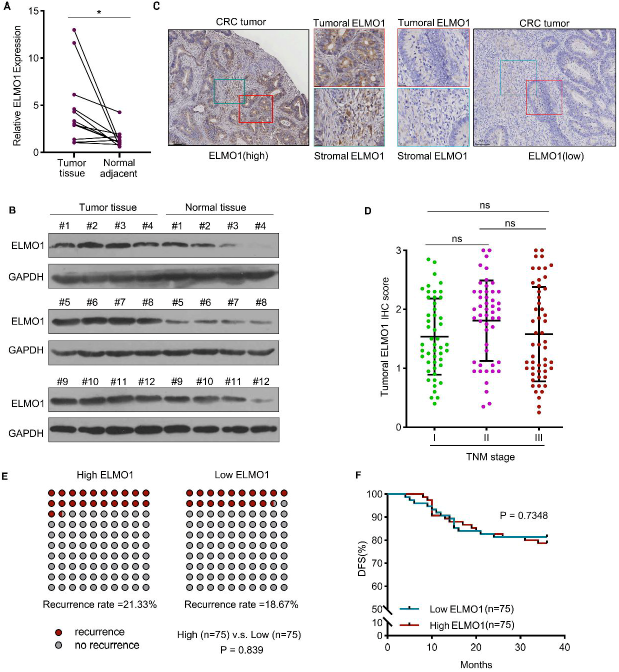


Colorectal cancer (CRC) is the third most common type of cancer worldwide, according to cancer statistics from 2021. There are an estimated 149,500 new cases of CRC and 52,980 CRC‐related deaths each year. While new therapeutics have significantly improved clinical outcomes, treating CRC after the cancer has migrated remains a challenge. This cancer pathogenesis through the body is facilitated by a type of white blood cell called tumor‐associated macrophage (TAM), which promote the proliferation, migration, and invasion of cancer cells. TAMs have potent tumor‐supportive behaviors. They are usually found suspended among the cells and other components of the tumor and comprise nearly 50% of the tumor mass.

Now, Wen et al. have identified how a protein called “engulfment and cell motility protein 1 (ELMO1)” plays a key role in the functional reprogramming of TAMs. The discovery sheds new light on tumor progression and provides new perspectives on improving clinical outcomes for patients with CRC. ELMO1 is known to be involved in cancer‐related cell signaling pathways, and in promoting malignancy in cancers affecting the brain, liver, and ovaries. However, the underlying molecular mechanisms behind how ELMO1 is involved in CRC development are not well‐understood.

The team collected 12 fresh and 150 preserved tissue samples from patients with CRC who had undergone surgical treatment at the Sixth Affiliated Hospital of Sun Yat‐Sen University. These samples were subjected to a series of tests, to identify the role of ELMO1‐reprogrammed macrophages in cancer development and migration.

Their findings revealed a much higher concentration of ELMO1 in TAMs, as compared to normal non‐malignant cells. Furthermore, ELMO1 was determined to reprogram macrophages via activating Rac1–a protein involved in cell proliferation, cell motility, and cell invasiveness. These ELMO1‐reprogrammed macrophages in turn promoted malignant activity of CRC cells, increased the production of tumor‐supportive cytokines, and drove cancer‐cell proliferation, migration, and invasion. On the other hand, inhibiting ELMO1 expression and regulating its levels promoted positive outcomes like reducing tumor‐supportive cytokine production.

This is the first study that comprehensively shows that ELMO1 reprograms macrophages into a TAM‐like phenotype, which ultimately feeds the progression of CRC. In the future, targeting rogue ELMO1 expression in TAMs could be an important therapeutic option for patients with CRC. https://onlinelibrary.wiley.com/doi/full/10.1111/cas.15628

## Proteolytic cleavage of membrane proteins by membrane type‐1 MMP regulates cancer malignant progression


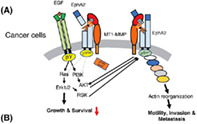


Components that surround and maintain the physical structure of animal cells are referred to as the extracellular matrix (ECM). Cancer cells degrade the ECM as they spread throughout the body. This degradation is aided by a family of enzymes known as matrix metalloproteinases (MMPs). A typical member of this enzyme family is the membrane‐type‐1 matrix metalloproteinase (MT1‐MMP), which contributes to the release and spread of cancer cells. Cancer therapies that inhibit the activity of MMP have been developed in the past, but have failed due to poor target specificity. To prevent cancer progression, new cancer therapeutics that specifically inhibit MT1‐MMP's ability to degrade membrane proteins are required.

In this issue, Ikeda et al. reviewed the mechanism by which MT1‐MMP breaks‐down cell membrane proteins and how the resulting fragments can be used to develop new cancer therapies and diagnostics in the future. To this end, they focused their study on the erythropoietin‐producing hepatoma receptor tyrosine kinase A2 (EphA2), a membrane protein that is strongly expressed in cancer cells. The findings revealed that the presence or absence of ligand‐bound receptors (EphrinA‐EphA2) influenced cancer progression via interactions with downstream molecules in the epidermal growth factor receptor (EGFR) signaling pathway. While the ligand‐free EphA2 receptor promoted cancer growth by interacting with EGFR signaling molecules, the ligand‐bound EphA2 receptor prevented cancer progression by inhibiting them.

They also described the clinical applications of two fragments (N‐terminal EphA2‐NF and C‐terminal EphA2‐CF) produced by fragmentation of EphA2 by MT1‐MMP. These fragments were found to be cancer‐specific and thus showed promise as therapeutic targets for cancer therapy. Since patients with pancreatic cancer have high levels of serum EphA2‐NF, they are currently being evaluated as a diagnostic indicator for this condition. EphA2 is also strongly expressed in aggressive hepatocellular carcinoma cells (HCCs) that are resistant to the drug, Sorafenib. However, because molecules involved in ligand‐free signaling have been linked to HCCs, EphA2‐CF is the preferred therapeutic target for HCCs over the unfragmented EphA2.

Overall, they discussed the potential for developing novel therapeutics targeting membrane proteins, which play an important role in cancer development and progression of malignant tumor cells activated by MT1‐MMP cleavage. https://onlinelibrary.wiley.com/doi/full/10.1111/cas.15638

